# The use of dose surface maps as a tool to investigate spatial dose delivery accuracy for the rectum during prostate radiotherapy

**DOI:** 10.1002/acm2.14314

**Published:** 2024-02-29

**Authors:** Haley M. Patrick, John Kildea

**Affiliations:** ^1^ Medical Physics Unit McGill University Montreal Quebec Canada

**Keywords:** dose delivery, dose reconstruction, prostate, radiotherapy, SBRT

## Abstract

**Purpose:**

This study aims to address the lack of spatial dose comparisons of planned and delivered rectal doses during prostate radiotherapy by using dose‐surface maps (DSMs) to analyze dose delivery accuracy and comparing these results to those derived using DVHs.

**Methods:**

Two independent cohorts were used in this study: twenty patients treated with 36.25 Gy in five fractions (SBRT) and 20 treated with 60 Gy in 20 fractions (IMRT). Daily delivered rectum doses for each patient were retrospectively calculated using daily CBCT images. For each cohort, planned and average‐delivered DVHs were generated and compared, as were planned and accumulated DSMs. Permutation testing was used to identify DVH metrics and DSM regions where significant dose differences occurred. Changes in rectal volume and position between planning and delivery were also evaluated to determine possible correlation to dosimetric changes.

**Results:**

For both cohorts, DVHs and DSMs reported conflicting findings on how planned and delivered rectum doses differed from each other. DVH analysis determined average‐delivered DVHs were on average 7.1% ± 7.6% (*p* ≤ 0.002) and 5.0 ± 7.4% (*p* ≤ 0.021) higher than planned for the IMRT and SBRT cohorts, respectively. Meanwhile, DSM analysis found average delivered posterior rectal wall dose was 3.8 ± 0.6 Gy (*p* = 0.014) lower than planned in the IMRT cohort and no significant dose differences in the SBRT cohort. Observed dose differences were moderately correlated with anterior‐posterior rectal wall motion, as well as PTV superior‐inferior motion in the IMRT cohort. Evidence of both these relationships were discernable in DSMs.

**Conclusion:**

DSMs enabled spatial investigations of planned and delivered doses can uncover associations with interfraction motion that are otherwise masked in DVHs. Investigations of dose delivery accuracy in radiotherapy may benefit from using DSMs over DVHs for certain organs such as the rectum.

## 1 INTRODUCTION

Interfraction motion of the prostate and rectum over the course of prostate radiotherapy is known to increase the risk of biochemical failure and rectal toxicities by altering delivered doses from planning intentions.^1^, ^2^ While image‐guided radiotherapy (IGRT) has been demonstrated to maintain prostate delivered doses within 3% of planning baseline, deviations in rectum delivered doses from planning may still exceed 20% for individual fractions and may, accordingly, lead to increased risk of rectal toxicities.^3^, ^4^, ^5^ These large deviations in daily rectum dose are especially of concern for prostate stereotactic body radiotherapy (SBRT) treatments, where each fraction heavily contributes to the total dose, making accurate fraction delivery important to ensure adequate rectum sparing. For this reason, it is important to quantify the level to which delivered and planned organ at risk (OAR) doses differ in clinical radiotherapy practice.

Comparisons of planned and delivered rectum doses have been conducted using 3D anatomical IGRT images in many previous studies.^6^, ^7^, ^8^, ^9^, ^10^, ^11^, ^12^ In nearly every study, daily delivered doses were calculated in treatment planning systems on IGRT images, with cone‐beam CT (CBCT) based studies employing additional voxel correction strategies (such as density overriding or deformed planning CTs) to account for the effects of differences in CBCT Hounsfield units (HU) on dose calculations.^7^, ^8^ Most previous studies investigated standard fractionated treatments using images from a subset of all treatment fractions and estimated total delivered rectum dose by averaging daily rectal dose‐volume histograms (DVHs),^6^, ^9^, ^10^, ^11^ which does not account for spatial dose changes between fractions. More recently, studies have begun to explicitly calculate total delivered dose distributions using deformable image registration (DIR) software to properly account for spatial dose variations in dose accumulation.^5^, ^8^, ^12^ However, despite the opportunity these 3D accumulated doses provide to investigate dose differences with full spatial context, researchers continue to use DVHs and other non‐spatial dose metrics for analysis.

DVHs have long been recognized to be limited by their lack of spatial information,^13^ making it possible for differences between dose distributions to be masked if they yield similar DVH metrics. One alternative to the DVH that preserves spatial information is the dose‐surface map (DSM), which provides a 2D representation of the dose to the surface of a structure. DSMs have become a popular tool for dose‐outcome studies of hollow organs, especially the rectum,^14^, ^15^, ^16^ and have recently been used to calculate total accumulated rectum dose for outcome studies of prostate radiotherapy.^17^, ^18^ There is limited research, however, investigating the potential benefits of using DSMs to compare planned and delivered rectal doses to evaluate radiotherapy delivery accuracy over DVHs, as well as a lack of DSM‐based studies of SBRT treatments.

The purpose of this study was to compare the ability of DVHs and DSMs to evaluate differences between planned and delivered rectum doses and to determine if DSMs offer an advantage over DVHs using real‐world data. Specifically, we compared planned and delivered doses for two independent patient cohorts using both DVHs and DSMs, and examined how our findings related to observed interfraction motion of the rectum and PTV.

## 2 METHODS

### 2.1 Patient cohorts

A retrospective single‐center patient cohort consisting of 20 patients with localized prostate cancer treated consecutively with SBRT starting from May 2020 were identified for this study. Patients in this cohort, henceforth referred to as the SBRT cohort, received 36.25 Gy in five fractions scheduled every‐other‐day. As it was also of interest to use DSMs to evaluate dose delivery accuracy for longer treatments, a second cohort of 20 patients with localized prostate cancer who were treated with IMRT to 60 Gy in 20 fractions between September 2015 and 2016 was also identified. This second cohort, henceforth referred to as the IMRT cohort, was used in a previous retrospective study^19^ and was conveniently selected due to its ready availability. While this was done to minimize the data preparation work required, the large time gap between IMRT and SBRT cohorts renders any comparisons between the two inappropriate and is therefore avoided in this work. Ethics approval for our retrospective study was granted by the Research Ethics Board (REB) of the McGill University Health Centre [project number 2024‐9506]. All work of the study was conducted in accordance with REB guidelines.

All patients underwent a simulation CT scan in a Philips Big Bore CT scanner using a 3 mm slice thickness. SBRT patients also underwent a T2‐weighted fast spin echo MRI simulation scan at 3T magnetic field strength to assist in prostate delineation. In preparation for simulation, all patients were instructed to empty their rectums and drink 500 mL of water thirty minutes before their imaging appointments. These instructions were repeated for each treatment fraction. No spacer gels or enemas were used in either cohort.

The target was defined as the prostate plus 7  or 5 mm isotropic margins for the IMRT and SBRT cohorts, respectively. OARs were contoured according to RTOG (Radiation Therapy Oncology Group) guidelines,^20^ beginning at the ischial tuberosities and finishing at the sigmoid junction. Volumetric modulated arc therapy (VMAT) plans consisting of two 6 MV arcs were designed for each patient in the Eclipse Treatment Planning System (Varian Medical Systems, Palo Alto, California, USA) in accordance with appropriate protocols: local published guidelines for the IMRT cohort,^21^ and NRG‐GU005 guidelines for the SBRT cohort.^22^

Treatments were delivered daily (IMRT) or every‐other‐day (SBRT) using CBCT IGRT guidance (resolution 0.9 × 0.9 × 2.0 mm^3^). For the IMRT cohort, the IGRT protocol used a single daily pre‐treatment CBCT to perform soft tissue matching before treatment delivery, whereas the SBRT cohort's IGRT protocol also mandated the acquisition of a daily post‐delivery CBCT to verify that no significant positional shifts had occurred during treatment. Additional pretreatment CBCTs were allowed for both cohorts in the event that large set‐up adjustments were required. Registrations between CBCTs and the planning CT were done at the treatment console and saved to the Oncology Information System.

### 2.2 Delivered dose calculation

Daily delivered doses were retrospectively calculated for each fraction in Eclipse using the patient's CBCT image for that fraction. To start, the registration of the patient's CBCT and planning CT were used to transfer the treatment beams to the last CBCT image acquired at each fraction. Next, couch and body structures were added. Full‐course prescription doses were recalculated on CBCT images using Eclipse's analytical anisotropic algorithm (v.15.6.06) and CBCT‐specific HU‐electron density curves obtained from measurements on each treatment linac in accordance with recommended best practices in the literature.^23^ These calibration measurements were performed using a 30 cm diameter, 18 cm deep cylindrical Cheese‐Phantom (Gammex RMI, Middleton, Wisconsin, USA) housing inserts representing a material density range of air to cortical bone. The accuracy of CBCT‐calculated dose distributions using these curves was evaluated in an anthropomorphic pelvis phantom planning study and determined to be within 1.15% of CT‐calculated plans, which agreed with previously reported values.^24^ We chose this method of daily‐delivered dose calculation over deformably registering planning CTs to daily anatomy due to the inability of our available clinical deformable registration software to meet TG‐132 recommended quality standards for rectum structures at the time of testing.^25^

To calculate daily rectal doses, rectum contours were retrospectively delineated on all daily CBCT images by a single observer to minimize interobserver contouring variations. Copies of the originally planned rectum contours were also reviewed and adjusted, if necessary, by the same observer to ensure retrospective contouring consistency across all patients. Finally, dose and contour data were exported as DICOM files from the treatment planning system to calculate DVHs and DSMs.

### 2.3 DVH and DSM calculation

Planned and daily rectum DVHs (recalculated to the full‐course prescription) for each patient were extracted from their DICOM‐RT dose files using a custom Python script. Total delivered doses for each patient were estimated by averaging daily DVHs to yield average‐delivered DVHs.

Generation of DSMs was achieved using *rtdsm*, an open‐source Python package previously developed by our group for DSM calculation and analysis.^26^ For each plan and daily image, *rtdsm* began by defining slices of the rectum contour orthogonal to its central‐axis path in increments of 3 mm, correcting the orientation of intersecting slices using the approach of Witztum et al.^27^ Next, 45 equiangular points were defined around the outer circumference of each slice and dose was sampled at these points. Finally, each contour was unwrapped along its posterior rectal wall and mapped into a 2D array. To calculate an accumulated DSM for each patient, their daily DSMs were aligned at their inferior borders and summed together, truncating longer daily DSMs to the length of the patient's shortest daily rectum contour. This approach was chosen to ensure daily rectal lumen doses were properly aligned with one another for summation as the inferior rectal border is less susceptible to interfraction changes than the superior border. This same DSM alignment and truncation approach was also used when calculating cohort DSM averages, limiting cohort‐wide comparisons of planned and delivered dose to the inferior‐most 10.2 cm of the rectum to match the length of the shortest observed rectum.

### 2.4 Comparison of planned and delivered doses

Planned and delivered DVHs and DSMs were compared on a patient‐by‐patient basis and cohort‐wide for both fractionation schemes. DVH differences were visualized by plotting planned and average‐delivered DVHs together with shaded regions around curves used to depict standard uncertainty of the mean across the treatment course or cohort. Dose differences between planned and delivered DSMs were visualized by calculating dose‐difference maps (DDMs).

Statistical comparisons of planned and delivered doses were conducted using permutation testing. For DVHs, tests were performed in steps of 5 Gy along the span of the full prescription dose range for each cohort (up 36.25 and 60 Gy, respectively). One‐sample tests were used to compare the distribution of each patient's daily‐delivered DVHs to their planned DVH and paired tests were used to compare planned and average delivered DVHs for each cohort. In order to compensate for the multiple comparisons problem, DVHs were only considered statistically different from each other if three or more consecutive testing points (i.e., a continuous 10 Gy range) yielded *p*‐values ≤ 0.05. DSMs were compared with multiple comparisons permutation (MCP) testing, which is a permutation testing technique designed for comparing 2D or 3D dose distributions like DSMs.^28^ As with DVHs, patient‐wise comparisons were performed using a one‐sample version of the test to compare the distribution of each patient's daily‐delivered DSMs (scaled up to prescription doses) to their planned DSM. Cohort‐wise comparisons used a paired version of the test to compare planned and accumulated DSMs for each treatment cohort. DSMs were considered statistically different from one another if five or more contiguous pixels yielded *p*‐values ≤ 0.05.

### 2.5 Influence of rectum and PTV inter‐fraction motion

As changes in rectum dose are frequently attributed to inter‐fraction changes in rectum size and shape, we investigated how these factors varied over the course of treatment to determine if the way in which they deviated from planning baselines could explain any dose differences observed. Three factors were evaluated: (1) change in rectal volume, (2) change in rectal wall position relative to the PTV, and (3) change in PTV position relative to the rectum inferior boundary. Planned and daily values for all three factors were obtained from the exported DICOM‐RT structure files. For rectal volumes, percent change from planning baseline was calculated for each patient to evaluate inter‐fraction change in rectum volume. Localized changes in rectal wall position were quantified by extracting point position information for the anterior and posterior rectum walls, as identified in the DSM calculation process, relative to the linac isocentre position for each day and compared to planning baseline. Lastly, change in the rectum location relative to the isocentre position was quantified by calculating the displacement vector between the isocentre and the centroid of the inferior‐most slice of the rectum for each day. The significance of deviations in rectal volume, wall positions, and PTV position from planning baseline was assessed using Wilcoxon signed‐rank testing (*p* < 0.05). Correlation between these factors and DVH/DSM dose was assessed using Pearson correlation. For DVHs, correlation analysis was performed for the V20%, V40%, V60%, V80%, and V100% metrics. For DSMs, correlation analysis was performed for six points along the anterior and posterior walls located 18, 36, and 54 mm from the inferior boundary. These points were selected as they were located in three regions of interest: directly below the level of the average PTV inferior margin, within the region of a notable DSM finding, and directly posterior to the centre of the PTV volume.

## 3 RESULTS

### 3.1 Comparison of planned and delivered dDoses

**FIGURE 1:**
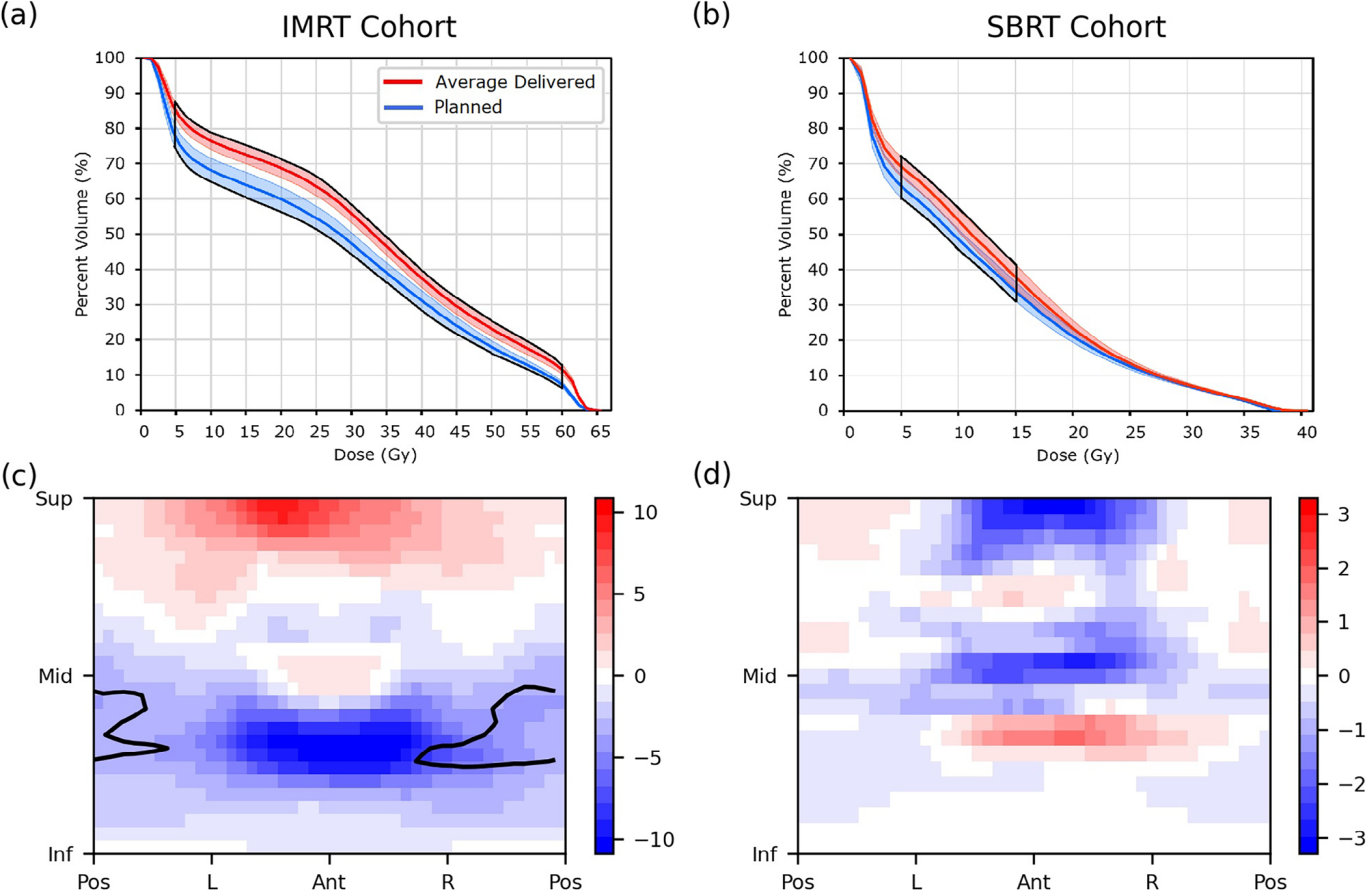
Average planned and delivered DVHs and cohort‐average dose difference maps for the IMRT (a, c) and SBRT (b, d) cohorts. Shaded regions around the DVHs represent standard uncertainty of the mean, and subregions with statistically significant dose differences are contoured in black.

DVHs and DSMs were found to yield different results on how planned and delivered doses differed for the two cohorts. As shown in Figure 1, DVHs indicated that averagedelivered rectal dose was statistically significantly higher than planned for the full prescription dose range in the IMRT cohort (*p* ≤ 0.002) and between 5 and 15 Gy for the SBRT cohort (*p* ≤ 0.021). Within these ranges, average‐delivered DVHs were on average 7.1% ± 7.6% and 5.0 ± 7.4% higher than planned DVHs for the IMRT and SBRT cohorts, respectively. In contrast, DSMs indicated that on average, patients in the IMRT cohort received statistically significantly lower doses than planned to a region of the posterior rectal wall (−3.8 ± 0.6 Gy, *p* = 0.014) and similar doses to what was planned to all other regions (Figure 1c). No statistically significant dose differences were observed using DSMs for the SBRT cohort in any region of the rectal wall (*p* = 0.411).

**FIGURE 2:**
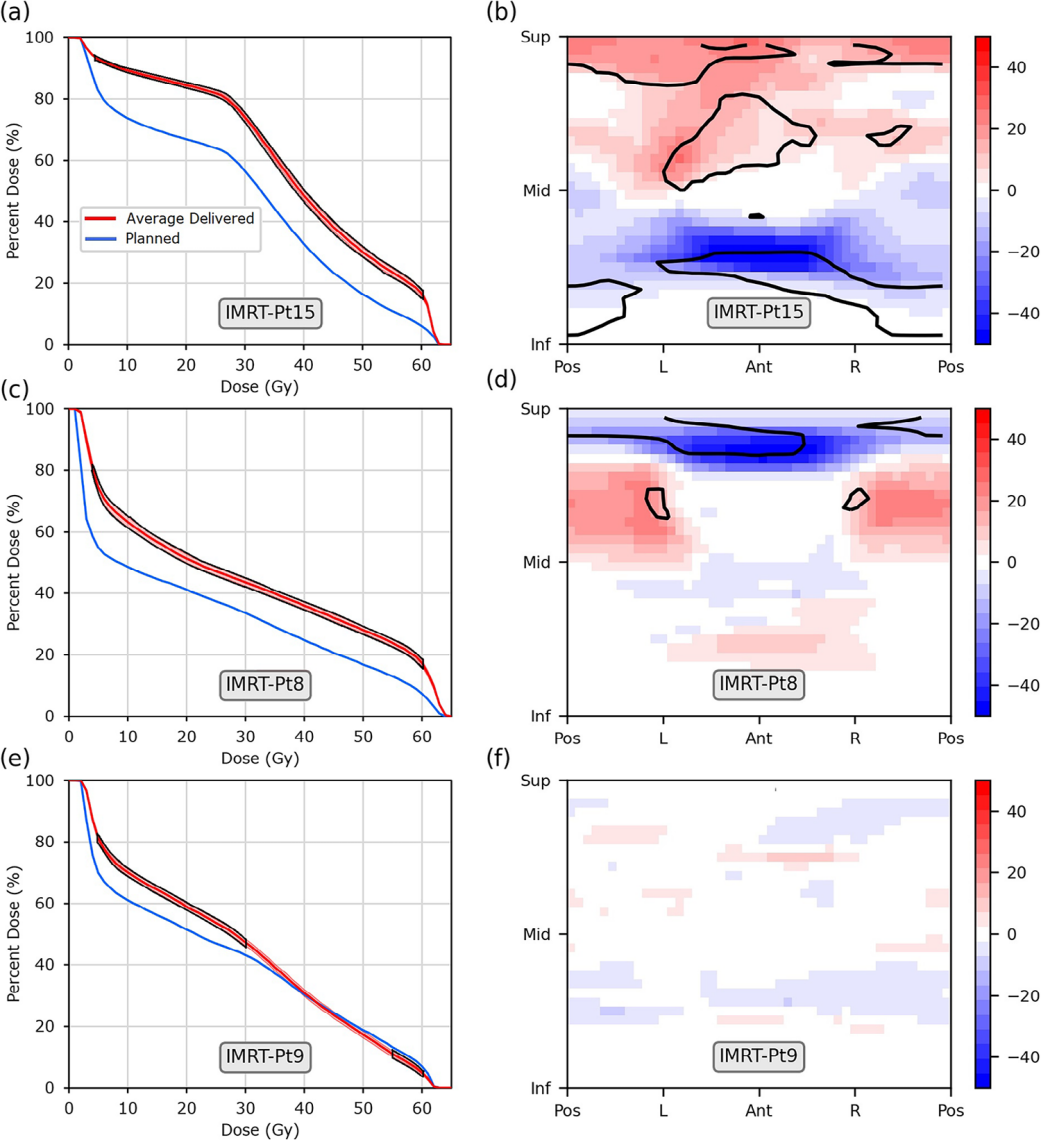
Example results for three patients from the IMRT (60 Gy in 20 fractions) cohort. (Left) Planned and average‐delivered DVHs and (Right) planned minus accumulated dose difference maps (DDMs), in units of Gy. Standard uncertainty of the mean for the average‐delivered DVH is depicted as the shaded region and subregions with statistically significant dose differences are outlined in black. Patients are identified by the grey labels.

**FIGURE 3:**
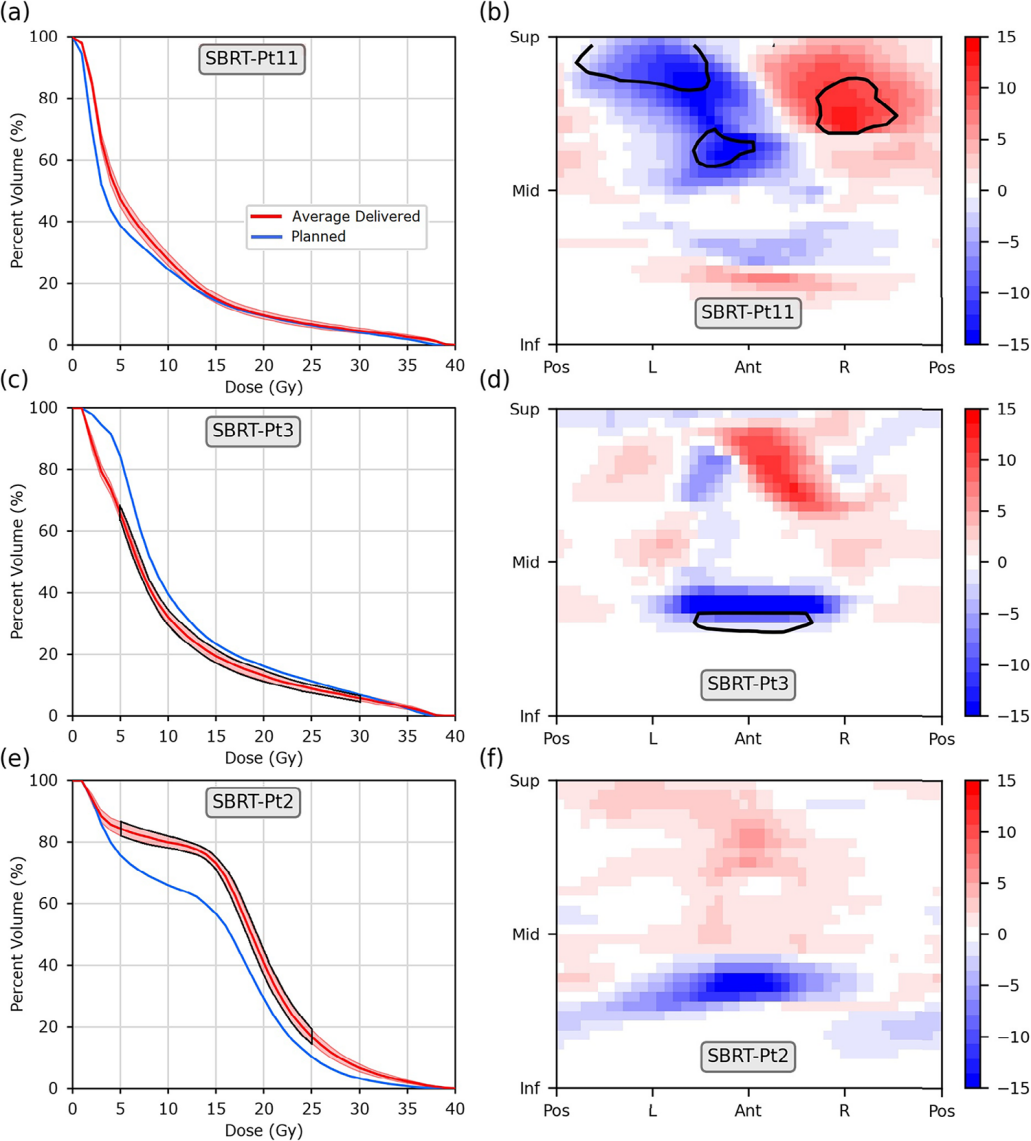
Example results for three patients from the SBRT (36.25 Gy in five fractions) cohort. (Left) Planned and average‐delivered DVHs and (Right) planned minus accumulated dose difference maps (DDMs), in units of Gy. Standard uncertainty of the mean for the average‐delivered DVH is depicted as the shaded region and subregions with statistically significant dose differences are outlined in black. Patients are identified by the grey labels.

Differing findings between DVH and DSM results persisted at the individual patient level, examples of which are shown in Figures 2 and 3. Data for all patients can be found in the Supplementary material. In total, 31/40 patients were identified to receive significantly different delivered doses from what was planned based on DVHs, whereas 18/40 were identified as such using DSMs.

Within the IMRT cohort, 20/20 and 12/20 patients were found to receive significantly different delivered doses from planned using DVHs and DSMs, respectively. While DVH‐based findings indicated that average‐delivered doses were higher for almost all individual patients, DSM‐based findings indicated most individual patients received higher doses in some regions of the rectum and lower doses in others (see Figure 2 for example).

Similar patterns were observed in the SBRT cohort. More patients were identified to receive significantly different average delivered doses than planned with DVH‐based findings (11/20) than with DSM‐based findings (6/20). DVHs found a majority of patients received higher delivered doses than planned. DSMs, however, only corroborated the DVH findings for three patients (patients 3, 9, and 19) while also identifying two patients to have significantly different planned and delivered doses that DVHs did not identify. Of note is Patient 11 (Figure 3a,b), for whom DVHs found no statistically significant dose differences despite exhibiting the largest regions of significantly different doses in their DDMs

### 3.2 Influence of rectal and PTV interfraction motion

**FIGURE 4:**
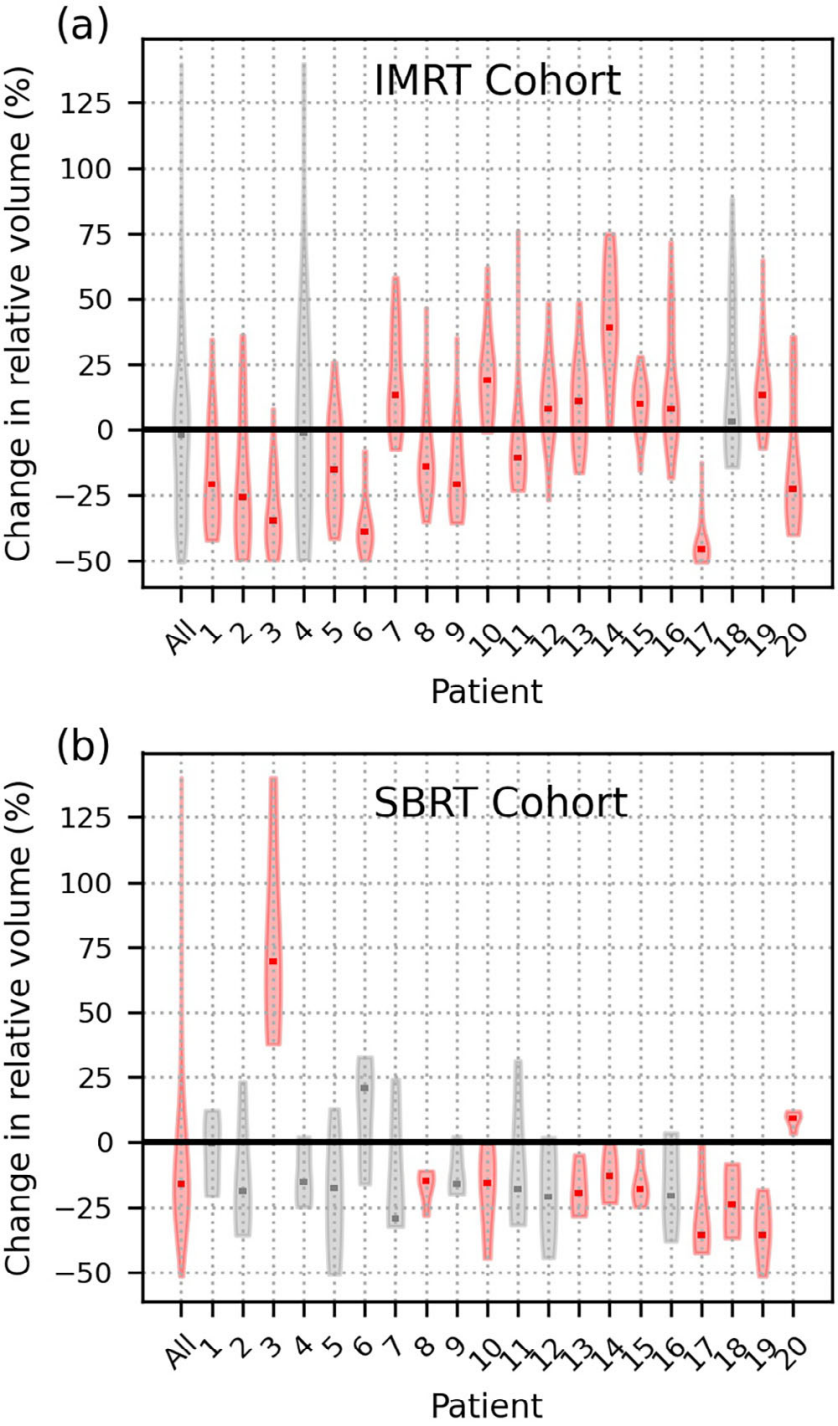
Violin plots of the relative change (planned minus delivered, relative to planned value) in daily rectal volume from planning value for the overall IMRT and SBRT cohorts (All), as well as individually numbered patients. Statistically significant results are indicated in red. Distribution medians are shown as rectangular markers.

**FIGURE 5:**
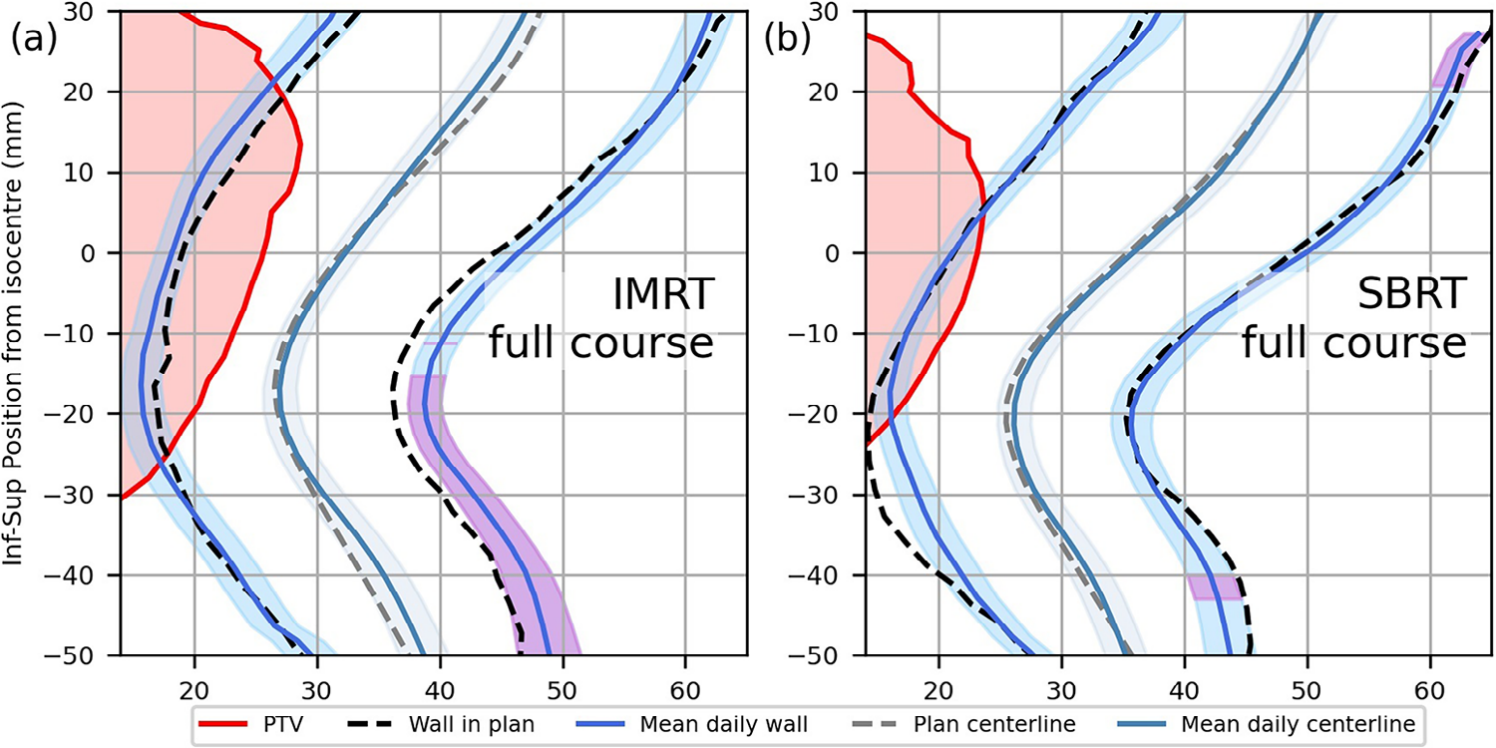
Mean rectal wall and PTV positions at planning and during treatment for the IMRT (a) and SBRT (b) cohorts, as well as their mean positions over the first (c) and last (d) five fractions for the IMRT cohort. Shaded regions indicate standard uncertainty of the mean, with purple areas indicating regions of statistically significant anterior‐posterior shifts.

Daily rectal volume variations were observed in all patients (Figure 4). In total, 18/20 IMRT and 10/20 SBRT patients had statistically significantly different rectum volumes during treatment compared to their planning volume. Interestingly, the SBRT patients tended to have smaller rectal volumes during treatment compared to at the time of planning, whereas no such cohort‐wide pattern existed for the IMRT patients. This volume‐reduction pattern is in line with what one would expect to yield hotter delivered DVHs than planned, as a smaller denominator (total rectal volume) will yield larger relative DVH values. However, it should be noted that overall correlation between rectal volume and rectal dose variations was negligible, as *R* ^2^ values were below 0.13 for all DVH and DSM metrics in both cohorts, suggesting other contributing factors may be responsible.Changes in rectal wall position between planning and delivery are shown for both cohorts in Figure 5. Rectal wall positioning was relatively stable throughout the course of treatment for the SBRT cohort, with significant shifts only observed for small segments of the posterior wall located inferior or superior to the level of the PTV (Figure 5b). Rectal wall positioning was much less consistent, however, for the IMRT cohort. On average, patients’ inferior‐posterior rectal walls were observed to have shifted significantly further from the target during treatment compared to their position at planning (Figure 5a). Within this region of statistically significant wall motion, the superior‐most pointsexperienced an average posterior shift of 5.5 ± 6.9 mm (*p* < 0.015) and corresponded with the region of significant dose difference (Figure 1c) seen in the DSMs. Moderate correlation was observed between posterior rectal wall position and dose within this region (*R* ^2 ^= 0.39), as well as for the region of wall directly posterior to the main PTV volume (*R* ^2 ^= 0.44). Within the SBRT cohort, moderate correlation was also observed between anterior or posterior rectal wall position and dose for the region directly posterior to the main PTV volume (*R* ^2^ = 0.48, 0.67, respectively). Anterior rectal wall motion in this region was also the factor most strongly correlated with change in any of the investigated DVH metrics, with *R* ^2^ values of 0.40 and 0.52 reported for the V80% metrics of the IMRT and SBRT cohorts, respectively.Table 1 presents the mean positional shifts of the PTV during treatment along each cardinal direction for both cohorts (shifts for each individual patient are shown in Table S1). A significant superior shift in PTV position (2.1 ± 4.2 mm) was observed for the IMRT cohort, with shifts of up to 11.1 mm observed for individual patients. This shifting of PTV position is discernable in the DDMs of several patients (such as Pt.15, Figure 2a) and the average DDM of the IMRT cohort (Figure 1c) as a band of increased dose to the superior rectum and band of decreased dose to the inferior rectum. Moderate correlation was observed between superior‐inferior PTV position and dose to the anterior and posterior rectal walls in the IMRT cohort (*R* ^2^ = 0.47, 0.46 respectively), but only for the region neighboring the average inferior PTV boundary. PTV position during treatment was more stable in the SBRT cohort, with an average superior shift of 0.3 (± 1.7) mm and maximum shift of 2.8 mm for an individual patient (Pt.2). Once again, DDMs corroborated this finding, as the same clear hot/cold banding observed in the IMRT cohort was not present in the DDMs of the SBRT cohort (Figures 1, 3). Correlation was weak to non‐existent between PTV motion and all dose metrics investigated (*R* ^2^ < 0.3).

**TABLE 1:** Average shifts in PTV position relative to the inferior rectum border for the IMRT and SBRT cohorts.

	Average shift from baseline (± St. Dev)	*p‐value*
IMRT Cohort: Left‐Right	0.3 ± 2.0 mm	0.416
IMRT Cohort: Ant‐Post	−1.6 ± 5.0 mm	0.063
IMRT Cohort: Sup‐Inf	2.1 ± 4.2 mm	**0.027**
SBRT Cohort: Left‐right	0.2 ± 1.8 mm	0.492
SBRT Cohort: Ant‐Post	1.1 ± 3.7 mm	0.143
SBRT Cohort: Sup‐Inf	0.3 ± 1.7 mm	0.245

Negative values correspond to the first listed direction, and positive values the second.

Bold values indicate p < 0.05.

## 4 DISCUSSION

The variation of daily delivered rectal dose over the course of prostate radiotherapy is a well‐documented phenomenon that can lead to different total delivered dose than what was planned. While rectal dose delivery accuracy is well‐characterized using DVHs, there is a current lack of studies performing the same analysis quantitatively with DSMs or other dose‐data representations that preserve spatial information. To address this, we quantified dose differences between planned and delivered rectum dose using DVHs and DSMs to determine what benefit, if any, the inclusion of spatial information adds.

In this study, we found that the way in which planned and delivered doses differ according to DSMs is substantially different from how they are found to differ using DVHs. For the cohorts studied, DVHs were more likely to indicate significant differences between planned and delivered doses, both for individual patients and at a cohort level, and to identify a singular way in which doses differed across the entire tested dose range (i.e., planned or delivered consistently higher or lower than the other). In contrast, DSM findings were more nuanced, frequently identifying spatial subregions that exhibited both significant increases and reductions in dose relative to planning intent. These findings are in line with a qualitative pilot study conducted by Scaife et al., who noted that accumulated DSMs identified regions with dose differences that went unindicated in accumulated DVHs.^29^ It is the only other study, to our knowledge, to evaluate dose delivery accuracy using planned and delivered DSMs, albeit in a more qualitative manner. We also note that our DVH findings are in line with previous studies of conventionally fractionated treatments. The rates at which we observed planned and average delivered DVHs to differ are similar to those reported by Hatton et al., who found 75−100% patients exhibited significant dose differences that were typically higher than planned,^11^ and Chen et al., who found 65−75% patients exceeded dosimetric constraints during delivery across the DVH metrics examined.^9^

In an effort to determine why our DVH and DSM findings differed, we investigated how rectal volumes and relative PTV and rectal wall positions changed over the course of treatment to try to discern if any patterns emerged. While changes in rectal doses have long been attributed to changes in rectal volume,^10^, ^30^ we did not observe clear evidence that rectal filling variations were a dominant influencing factor in our dosimetric findings. Although systematically reduced rectal volumes during treatment in the SBRT cohort did provide a plausible explanation for the significantly higher mean *V*_5Gy—_ *V*_15Gy_ values observed, little to no correlation between rectal volumes and DVH/DSM metrics existed. Instead, we found that changes in rectal wall position better explained the dosimetric differences we observed, as they were moderately correlated with DVH V80% and localized DSM dose variations. Changes in PTV location, relative to the rectum, were also moderately correlated with anterior and posterior rectal wall dose at the level of the inferior PTV margin in the IMRT cohort. Overall, we found correlations were stronger between interfraction motion metrics and DSM data than motion and DVH metrics. We suspect that this may be due to the preservation of spatial context in DSM dose data allowing for more straightforward correlations to be assessed. For example, it is fairly intuitive that a significant shift in posterior rectal wall position away from the PTV would yield a significant reduction in posterior wall dose, or that a superior‐inferior shift of the PTV (and therefore the dose‐wash distribution) would most affect rectal dose to points near the PTV superior and inferior boundaries. In contrast, it is much less intuitive how changes such as these will affect a DVH metric, as the metric is sensitive to volume and positional changes across the entire rectum and not just those in one region of interest.

The advantages of using DSMs instead of DVHs have been previously demonstrated for dose‐outcome investigations.^14^, ^15^, ^18^ This study further expands on the benefits of using DSMs over DVHs by demonstrating their usefulness to identify spatial dose deposition differences between what is planned and delivered. As we have shown, DSM's preservation of spatial dose information can enable the identification of spatial dose deposition patterns that are indicative of positional shifts of the rectum and PTV, such as the red‐blue banding pattern in DDMs indicative of superior‐inferior PTV shifts (Figures 1, 2). For this reason, DSMs could be useful for studies of motion mitigation interventions by allowing for a more detailed analysis of the dosimetric impact of an intervention on treatment delivery accuracy. This analysis may also be useful for clinical trials to verify planned dose distributions are sufficiently similar to delivered dose distributions and estimate the uncertainty introduced by dose deviations on study conclusions. It may also be useful on an individual patient basis to evaluate if dose delivery accuracy may have contributed to the development of rectal toxicities.

Although our study provides insight on the different capabilities of DVHs and DSMs to identify variations between planned and delivered dose, we recognize there are several limitations to it. One of the most important is that we lacked access to a deformable image registration software that performed to TG‐132 quality standards,^25^ leading us to calculate CBCT delivered doses and accumulated doses with non‐deformable registration methods. While the colloquially suggested best practice for dose recalculations on CBCT anatomy is to perform dose calculations on CT images deformed to the CBCT anatomy, sufficient evidence to definitively conclude this method is superior to others is lacking.^24^ Furthermore, we ensured we followed best practices when calculating dose directly on CBCTs, using a Gammex phantom of sufficient diameter and depth to properly recreate the scatter contributions expected from pelvic CBCT acquisition when measuring our CBCT‐specific HU‐electron density curves,^23^ which should ensure our dose calculations are within 2% of CT‐based calculations.^24^ However, we recognize that the average‐delivered DVHs used in this study may not accurately represent true delivered DVHs that would be better estimated using deformable dose accumulation strategies,^31^ and therefore stress our findings only apply to average daily delivered dose. Delivered DSMs, on the other hand, are expected to be much closer to true total delivered doses as they have been demonstrated to accurately accumulate rectum delivered doses in experimental measurements.^32^ Additional limitations to our study are the possible influences of intra‐observer contouring variations on rectum contours, and subsequently, dose, as well as a lack of consideration for intra‐fraction motion in delivered dose calculations and our inability to compare the IMRT and SBRT cohorts. Based on previous literature, we estimate the potential dosimetric uncertainty introduced by intra‐observer contouring variations to be within 3%^33^ and negligible effects (< 1%) from intrafraction motion.^34^

## 5 CONCLUSION

DSMs are capable of detecting complex dose delivery variations to the rectal wall indicative of different types of interfraction motion that are not discernable from DVH data. Future investigations of dose delivery accuracy should consider moving away from non‐spatial dose metrics like DVHs and towards spatial dose comparison tools like DSMs.

## AUTHOR CONTRIBUTIONS

Haley M. Patrick designed the study, prepared and processed the data, performed the analysis, and wrote the manuscript. John Kildea contributed to the study design, data interpretation, and reviewing the manuscript.

## CONFLICT OF INTEREST STATEMENT

No conflicts of interest.

## Supporting information

Supporting Information
