# Supplementary material for: The use of dose surface maps as a tool to investigate spatial dose delivery accuracy for the rectum during prostate radiotherapy
Source: J Appl Clin Med Phys. 2024 Feb 29;25(7):e14314. doi: 10.1002/acm2.14314 (PMC11244681; doi:10.1002/acm2.14314)
Supplement: Supplementary file 1 — Supporting Information [file ACM2-25-e14314-s001.pdf]

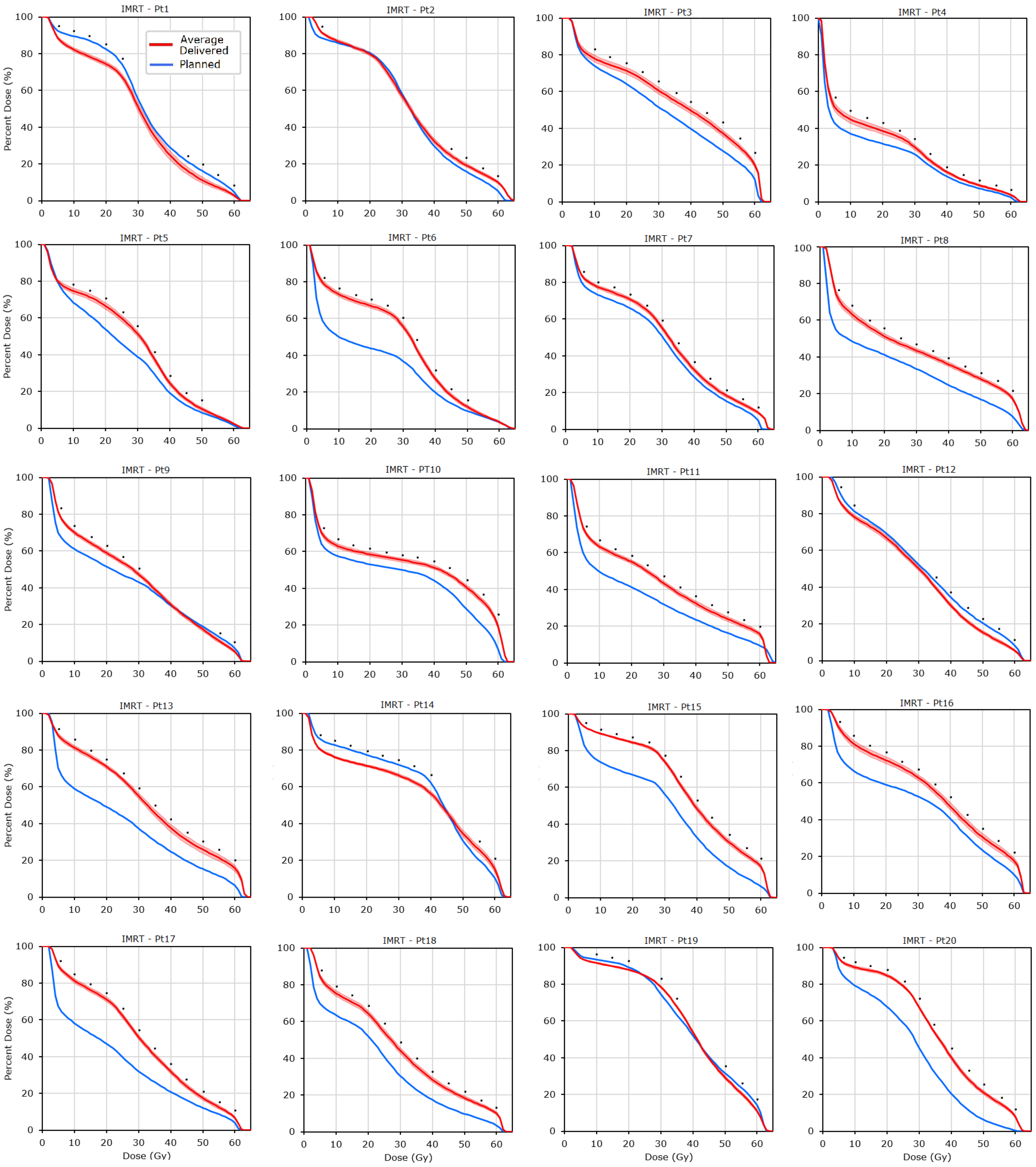

Figure S1: Planned and average-delivered DVHs of the IMRT treatment patients. DVH metrics with statistically significant differences between planned and average-delivered doses are indicated with black dots. Only DVHs with three or more coinciding statistically different DVH metrics were considered to be statistically different for the purposes of this paper.

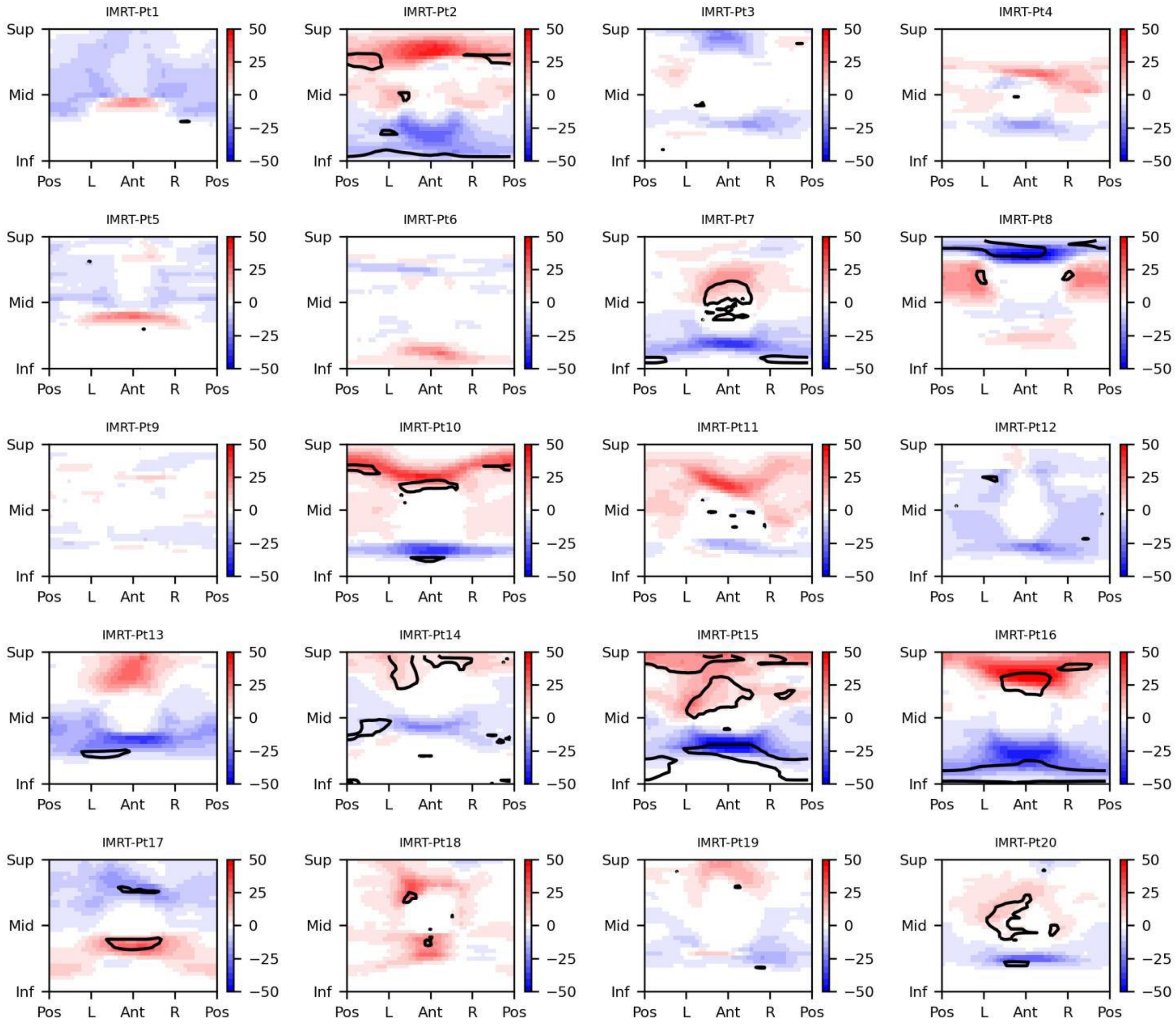

Figure S2: Dose difference maps (DDMS) of the IMRT treatment patients, in units of Gy. Subregions with statistically significant dose differences are contoured in black. Only subregions consisting of five or more continuous pixels were considered as statistically significant subregions for the purposes of this paper.

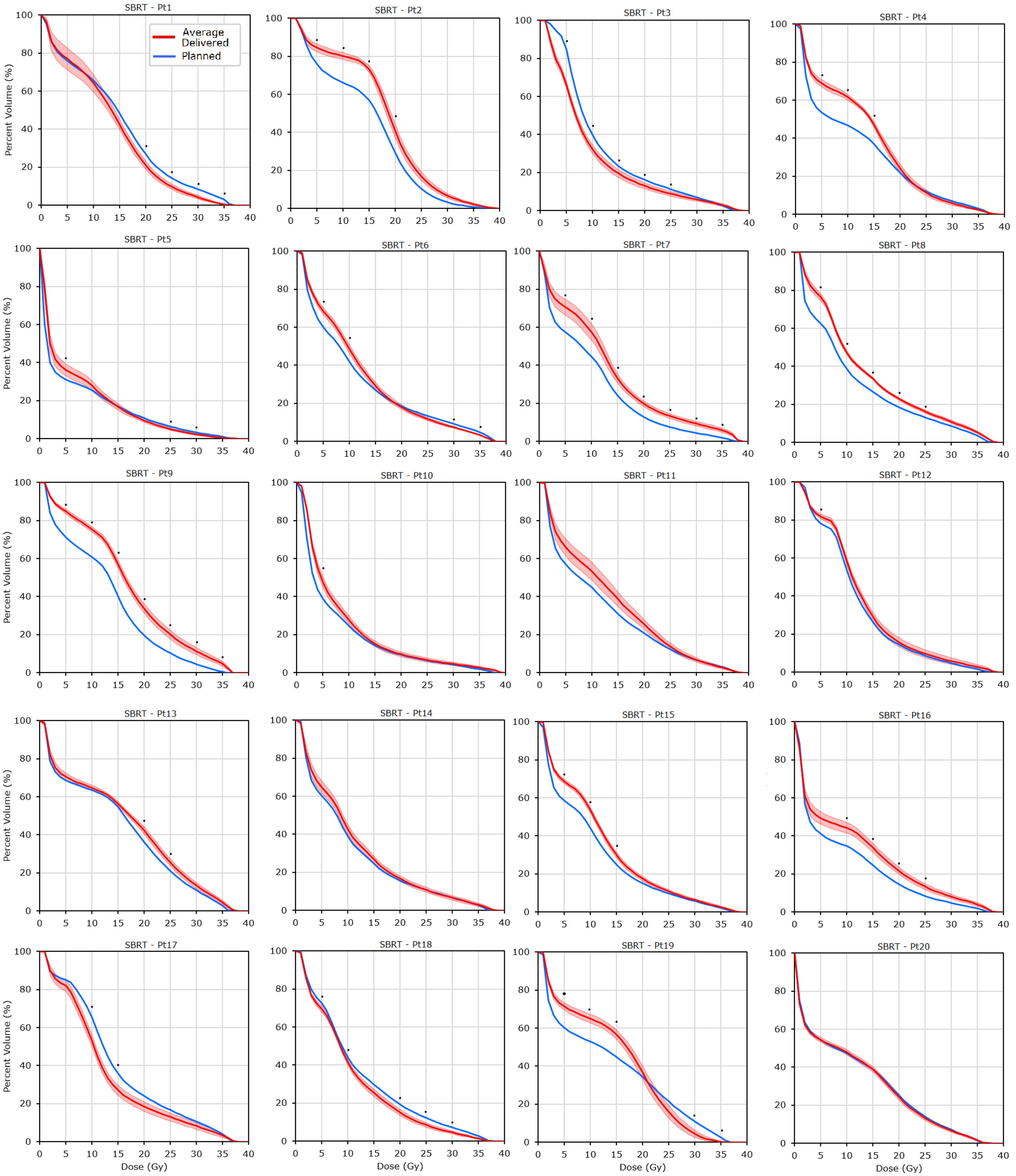

Figure S3: Planned and average-delivered DVHs of the SBRT treatment patients. DVH metrics with statistically significant differences between planned and average-delivered doses are indicated with black dots. Only DVHs with three or more coinciding statistically different DVH metrics were considered to be statistically different for the purposes of this paper.

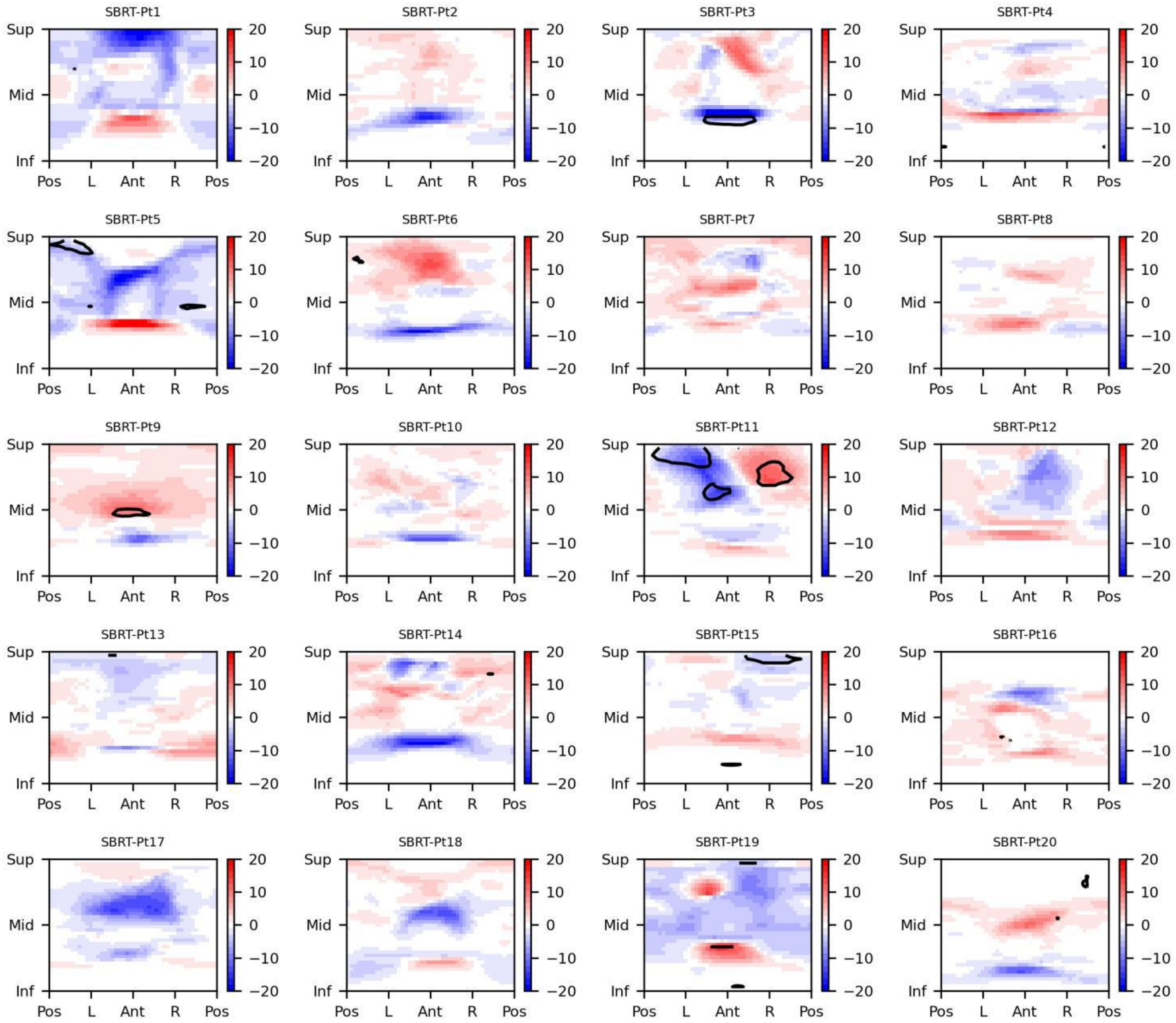

Figure S4: Dose difference maps (DDMS) of the SBRT treatment patients, in units of Gy. Subregions with statistically significant dose differences are contoured in black. Only subregions consisting of five or more continuous pixels were considered as statistically significant subregions for the purposes of this paper.

Table S1: Average positional shifts of the prostate PTV relative to the rectum inferior boarder between planning and delivery for each individual patient. Negative values correspond to a shift in the first listed direction and positive values the second.

	Average L-R Shift from Planning Baseline (mm)	Average Ant-Post Shift from Planning Baseline (mm)	Average Sup-Inf Shift from Planning Baseline (mm)
**IMRT-Pt1**	-1.37	-8.66	-2.04
**IMRT- Pt2**	-0.70	-3.01	8.46
**IMRT- Pt3**	2.60	-3.83	-0.51
**IMRT- Pt4**	0.14	-4.95	0.08
**IMRT- Pt5**	-0.85	0.50	-0.07
**IMRT- Pt6**	-0.57	0.22	-1.14
**IMRT- Pt7**	0.20	-2.93	6.19
**IMRT- Pt8**	-2.62	2.00	-1.95
**IMRT- Pt9**	0.00	-2.96	-0.88
**IMRT- Pt10**	-1.59	7.43	4.78
**IMRT- Pt11**	-0.05	1.08	0.33
**IMRT- Pt12**	2.34	-4.80	1.75
**IMRT- Pt13**	-0.25	-2.38	5.08
**IMRT- Pt14**	0.82	-5.15	0.77
**IMRT- Pt15**	-0.68	-1.39	8.47
**IMRT- Pt16**	-2.81	-1.36	11.09
**IMRT- Pt17**	-1.73	3.91	-1.04
**IMRT- Pt18**	0.79	2.05	-1.38
**IMRT- Pt19**	0.52	-4.58	2.50
**IMRT- Pt20**	0.60	-4.08	1.58
**SBRT-Pt1**	0.42	1.34	0.19
**SBRT- Pt2**	0.23	-0.35	2.84
**SBRT- Pt3**	-1.22	1.26	1.07
**SBRT- Pt4**	-4.41	0.39	0.88
**SBRT- Pt5**	-0.98	3.26	-0.89
**SBRT- Pt6**	0.48	-5.45	-0.51
**SBRT- Pt7**	-2.12	6.46	2.25
**SBRT- Pt8**	1.52	1.08	-0.03
**SBRT- Pt9**	0.98	4.43	0.81
**SBRT- Pt10**	0.64	3.39	0.50
**SBRT- Pt11**	0.55	1.31	-0.06
**SBRT- Pt12**	-0.59	4.85	-1.37
**SBRT- Pt13**	0.16	0.97	-0.54
**SBRT- Pt14**	-0.96	-2.89	2.02
**SBRT- Pt15**	0.40	4.11	-1.34
**SBRT- Pt16**	1.02	1.25	-1.39
**SBRT- Pt17**	-0.49	-3.51	-1.23
**SBRT- Pt18**	-0.56	3.24	1.47
**SBRT- Pt19**	1.65	-1.75	0.54
**SBRT- Pt20**	-1.09	-2.33	1.65
